# Custom 3D-printed implants for severe acetabular bone loss in revision total hip arthroplasty: A review of current advances and future directions

**DOI:** 10.1051/sicotj/2026062

**Published:** 2026-10-08

**Authors:** Ashish Singh, Purushotam Kumar, Sudhir Shankar Mane, Himanshu Swarnkar, Kanukuntla Kalyan, Akash Chandrashekar Gundalli, Lokesh Goyal, Jithin M. Vipin, Rabindra Narain Singh

**Affiliations:** Department of Orthopaedics, Anup Institute of Orthopaedics & Rehabilitation Patna Bihar India

**Keywords:** Revision total hip arthroplasty, Acetabular bone loss, 3D-printed acetabular implants, Paprosky classification, Aseptic loosening, Custom implants

## Abstract

Massive acetabular bone loss in revision total hip arthroplasty (rTHA) presents a major reconstructive challenge, particularly in Paprosky type III defects and pelvic discontinuity. Conventional reconstructive strategies, including biologic grafting, porous metal augments, and cup-cage constructs, may not adequately address complex three-dimensional (3D) defect morphology or achieve durable fixation in severe bone loss. This narrative review summarises current reconstruction strategies, with particular emphasis on the evolution, biomechanical principles, clinical outcomes, and future directions of custom 3D-printed acetabular implants. A literature search of PubMed, Google Scholar, and Embase (via Ovid) identified relevant clinical studies, systematic reviews, and registry analyses addressing reconstruction of severe acetabular defects using custom 3D-printed acetabular implants. Current evidence demonstrates favourable short- to intermediate-term clinical and radiographic outcomes, with reported implant survivorship of 95.8% at 3–6 years and 95% at 10 years in selected series. Compared with conventional modular reconstruction, custom 3D-printed acetabular implants improve conformity to complex defects, facilitate restoration of the hip centre of rotation, and optimise implant–bone contact and fixation. Reported complications are generally of lower rates comparatively, with postoperative dislocation, periprosthetic joint infection, and sciatic nerve palsy being the most frequently described adverse events, although the available evidence remains largely limited to retrospective case series with heterogeneous implant designs and relatively short follow-up. Two institutional cases are presented as illustrative examples of CT-based planning, custom implant manufacture, and surgical application in severe Paprosky type III acetabular defects. Custom 3D-printed acetabular implants represent an important advancement when conventional biologic and modular reconstruction techniques are insufficient. Future developments in artificial intelligence-assisted planning, robotic guidance, and sensor-enabled implants may further improve implant design, surgical precision, and postoperative monitoring; however, prospective comparative studies with longer follow-up are required to establish long-term durability and comparative effectiveness.

## Introduction

Revision total hip arthroplasty (rTHA) is an increasingly common and resource-intensive procedure, reflecting the global rise in primary joint replacement surgeries and improved life expectancy. Among the leading indications for revision are aseptic loosening, instability, prosthetic joint infections (PJI), and periprosthetic fracture [1]. In late implant failure, aseptic loosening remains the predominant cause, and a major challenge in the revision process is acetabular bone loss (osteolysis).

Severe acetabular defects present a distinct reconstructive challenge. Extensive osteolysis compromises the structural integrity of the acetabulum, disrupts normal load transfer across the pelvis, and alters the hip centre of rotation (COR) [2]. Achieving durable fixation in the presence of limited host bone is technically demanding, particularly in Paprosky type III defects and cases approaching pelvic discontinuity (PD).

Multiple reconstructive strategies have evolved, ranging from biologic graft-based techniques to modular prosthetic systems and, more recently, patient-specific custom implants. This review discusses the previous and current reconstructive strategies for severe acetabular bone loss, highlights the biomechanics and clinical applications of custom 3D-printed acetabular implants. We present selected institutional examples of the components in use for rTHA, and finally, we discuss potential directions for future research in this domain.

## Methods

A manual search of the databases of PubMed, Google Scholar, and Embase (via Ovid) to identify relevant studies addressing the reconstruction of severe acetabular defects in rTHA. Search terms included combinations of “acetabular bone loss,” “revision total hip arthroplasty,” “pelvic discontinuity,” “acetabular defects,” “custom-made implants”, “Paprosky type III,” and “custom 3D-printed acetabular implant”. Original articles, case series, systematic reviews, and registry analyses published in English were reviewed. Studies were selected based on relevance to severe acetabular defects and reconstruction strategies employed. The results are presented by theme, focusing on current reconstruction strategies, with an emphasis on custom 3D-printed acetabular implants, clinical evidence, and future directions in reconstructive orthopaedic surgery.

## Classification and biomechanical implications of acetabular bone loss

Accurate classification of acetabular defects is essential for surgical planning. The Paprosky classification, which is based on radiographs, remains widely used and stratifies defects based on the evaluation of four radiographic criteria: migration of the hip rotation centre, ischial osteolysis, teardrop osteolysis, and disruption of the Kohler line, with the most challenging type being Type IIIB [3]. Recently, a 3D-based classification has been proposed, though its widespread implementation might be limited due to the need for more technical expertise [4].

In type III defects, substantial superior migration of the acetabular component reflects loss of supportive bone and altered force transmission. Disruption of column support leads to increased shear stresses and eccentric loading. Restoration of the hip COR becomes critical to re-establish abductor mechanics and reduce abnormal joint forces [3]. When managing acetabular defects, three objectives are important, which include: 1) secure acetabular fixation with maximal host-bone contact and limited micromotion, 2) optimal limb length, hip biomechanics, and joint stability, and 3) host bone preservation.

Understanding these biomechanical implications guides implant selection and fixation strategy. Reconstruction strategies in rTHA are guided by the degree of available bone stock, the presence of PD, patient factors, and the overall defect pattern. Broadly, current options can be categorised into biologic reconstruction techniques, modular prosthetic solutions, and custom implants.

### Biologic reconstruction

Early management of massive acetabular defects focused on biologic reconstruction using bulk structural bone grafting and impaction bone grafting.

Bulk bone allografting, often utilising a femoral head allograft, was intended to restore bone stock while providing structural support and bony integration. However, this often required wide exposure on the ilium, with significant risk to the superior gluteal neurovascular structures, and long-term survival was inconsistent, with reported graft resorption rates of around 35% in some series [5, 6]. Progressive graft collapse, infection and loosening limited its durability in rTHA, particularly in larger segmental defects [7].

Impaction bone grafting involves morselised graft supported by reinforcement rings or meshes. It demonstrated favourable outcomes, including good bone healing in small, contained medial defects, when strict postoperative protocols were followed. However, when applied to larger segmental defects such as Paprosky type III defects, the availability of an adequate amount of graft, graft resorption, and mechanical failure were major challenges associated [8].

Acetabular distraction was later developed specifically to address PD – independent motion of the superior and inferior segments. This technique relies on ligamentotaxis to create a pseudo-press-fit construct for cups/augments and to promote osseointegration. While early stability can be achieved, cup migration and micromotion have been reported, potentially compromising long-term fixation [9]. As a result, these biologic approaches gradually declined as more stable prosthetic solutions emerged.

### Modular prosthetic solutions

The introduction of highly porous trabecular metal has substantially changed reconstruction strategies for acetabular bone loss in rTHA. Porous metal implants provide high frictional resistance, a structural similarity to cancellous bone, and a surface architecture that supports osseointegration usually seen within the 14 ± 2 months follow-up assessment. These properties provide mechanical stability and act as prosthetic substitutes to structural allografts.

Unlike structural allografts, they are not susceptible to graft resorption, collapse, or disease transmission. They are available in several shapes and sizes and can be used to fill segmental defects, support a hemispherical cup, and improve contact between the reconstruction and viable host bone. However, as modular off-the-shelf components, they may not fully conform to highly irregular defects and may require additional bone resection to achieve adequate seating, limiting their success in achieving stable fixation and sufficient host-bone contact.

Cup-cage constructs were developed for more extensive defects, such as Paprosky Type IIIA and IIIB defects and PD. These constructs combine a porous metal acetabular shell, which provides the potential for biologic fixation, with an antiprotrusio cage that bridges the defect and transfers the load to stronger pelvic regions. Chang et al. [10] reported acceptable intermediate-term outcomes, with survivorship around 82.1%; however, outcomes are less predictable in high-grade acetabular bone defects and PD, where failure rates increased from 14.9% to 40% depending on defect severity. [11]

The main limitation of cup-cage reconstruction is that long-term durability depends on osseointegration of the porous cup rather than biological fixation of the cage itself. The cage provides initial mechanical support, but its flanges are not designed for substantial bone ingrowth. Failure may therefore occur if the porous shell does not achieve adequate osseointegration, if fixation is limited by poor host bone, or if the construct is subjected to persistent micromotion across a large defect or PD. Reported complications include instability, sciatic nerve injury, intraoperative fracture, infection, aseptic loosening, and further revision, particularly in patients with poor bone quality or severe segmental defects [8, 10, 11]

Overall, modular porous metal augments and cup-cage constructs have improved fixation compared with earlier biologic reconstruction techniques and remain valuable options in selected cases, such as Paprosky Type I and II defects. However, their standardised geometry limits their ability to match highly irregular three-dimensional (3D) defects. This limitation provides the rationale for patient-specific 3D-printed acetabular implants, which are designed to conform to the individual defect, optimise host-bone contact, and pre-plan fixation trajectories.

### Custom implants

Custom implants represent a recent evolution in complex acetabular reconstruction. Traditional off-the-shelf implants are limited in this setting because complex, irregular defects can reduce implant-bone contact, compromise initial fixation, and limit osseointegration [12]. Custom 3D-printed implants address these limitations by manufacturing a porous titanium implant with an elasticity and microstructure similar to bone, conforming to the individual defect anatomy. This approach can improve implant–bone contact, optimise screw trajectories, and enhance construct stability in large defects and PD [7].

A major advantage of custom 3D-printed acetabular reconstruction is the ability to accurately plan the implant position around the intended anatomical COR compared to traditional implants. Accurate COR restoration is central to the rTHA because it influences limb length, hip stability and implant survivorship [13]. Early clinical series support the accuracy of 3D implants: Goriainov et al. (2021) [13] and Fang et al. (2022) [14] reported postoperative vertical and horizontal COR deviations of ≤2 mm after reconstruction with custom 3D-printed acetabular implants. This is clinically important because medial or superior displacement of the COR reduces abductor efficiency and may contribute to increased rates of revision. Conversely, inferior displacement creates significant tension in the gluteus medius muscle and increases tension on adjacent neurovascular structures, including the sciatic nerve, potentially causing pain or neuropathic symptoms [15].

Although this review primarily addresses complex revision arthroplasty, the principles underlying patient-specific acetabular reconstruction are equally applicable to other forms of severe acetabular deficiency, particularly developmental dysplasia of the hip (DDH), where traditional implants may not achieve adequate fixation due to deficient bone stock [16]. Evidence from primary THA in DDH further supports this concept. Chen et al. [16] demonstrated that patients treated with custom 3D-printed implants experienced significantly lower intraoperative blood loss, earlier mobilisation, shorter hospital stay, improved acetabular cup coverage and higher Harris Hip Scores (HHSs) during the first postoperative year compared with the non-3D group; thus reinforcing the broader principle that defect-specific implant design and accurate preoperative planning can improve anatomical reconstruction and early functional recovery.

Current evidence suggests favourable implant survival rates. A review by Malahais et al. [17] and Guo et al. [15] reported an overall survivorship of 95.8%–100% at 3–6 years, with a revision rate of only 4.2%, comparing favourably with the 91.9% survivorship reported for cup-cage constructs in similar bone defects in the former study. Longer-term data also appear encouraging; Meding et al. [18] reported a 10-year survivorship of 95% (38 out of 40 implants) with a 7.5% reoperation rate. Across published studies, complication rates following custom 3D-printed acetabular reconstruction have generally been comparable to or slightly lower than those reported for other reconstructive strategies. The most frequently reported complications include postoperative dislocation, periprosthetic joint infection, and sciatic nerve palsy, with dislocation remaining the predominant cause of reoperation [15, 17, 18].

### Illustrative case series

Preoperative planning begins with thin-slice computed tomography, using metal artefact reduction protocols, to accurately assess residual host bone. Segmentation of the hemipelvis allows generation of a high-fidelity 3D model, allowing assessment of defect morphology, bone thickness, and remaining structural support. Virtual reconstruction enables restoration of the anatomical COR, simulation of acetabular reaming and pre-planning screw trajectories into the ilium, ischium, and pubis while avoiding intersecting paths. Porous trabecular titanium surfaces are incorporated to enhance osseointegration and biological fixation.

Our institutional experience illustrates the application of this workflow in two patients with Paprosky Type IIIA defects, one of whom had previously undergone cage reconstruction. The index patient, a 41-year-old male who had undergone cemented THA 14 years earlier for avascular necrosis of the femoral head, presented with progressive mechanical hip pain, significant limb-length discrepancy, and inability to bear weight that had developed over the past few years. Radiographs demonstrated superior migration of the acetabular component, disruption of Kohler's line, and extensive periacetabular osteolysis. CT imaging confirmed substantial bone loss involving the acetabulum, ischium, pubis, and proximal femur. Formal PD was not evident, and the defect was classified as Paprosky Type IIIA (Figure 1) rather than Type IIIB.

**Figure 1:**
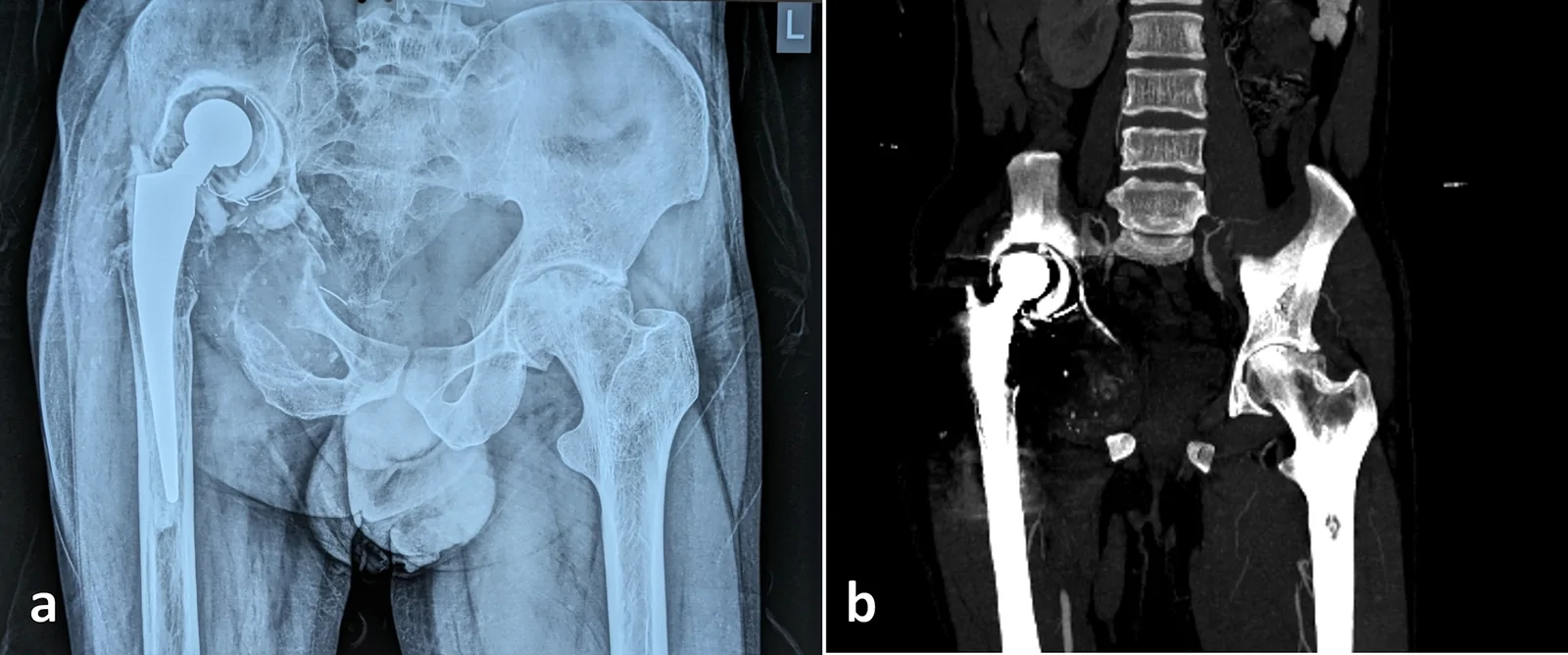
Imaging of the pelvis. (a) Anteroposterior view radiograph of the pelvis showing proximal migration of the acetabular cup and femoral stem into the ilium and disruption of Kohler's line; (b) Computed tomography image of the pelvis and proximal femur showing proximal migration of the hip prosthesis and femoral bone loss.

Following CT-based modelling and segmentation using *MySegmenter* software (Jajal Medical Services, Vadodara, India), a custom porous titanium acetabular component (Jajal Medical Services, Vadodara, India) was designed to match the irregular anatomy of the defects. Multiple locking screw options were incorporated, and trajectories were predefined to optimise fixation. The custom 3D-printed acetabular implant was manufactured using additive manufacturing techniques and delivered within ten days (Figure 2). A physical 3D-printed pelvic model made of polylactic acid (PLA) was used for surgical rehearsal and intraoperative reference (Figure 2d).

**Figure 2:**
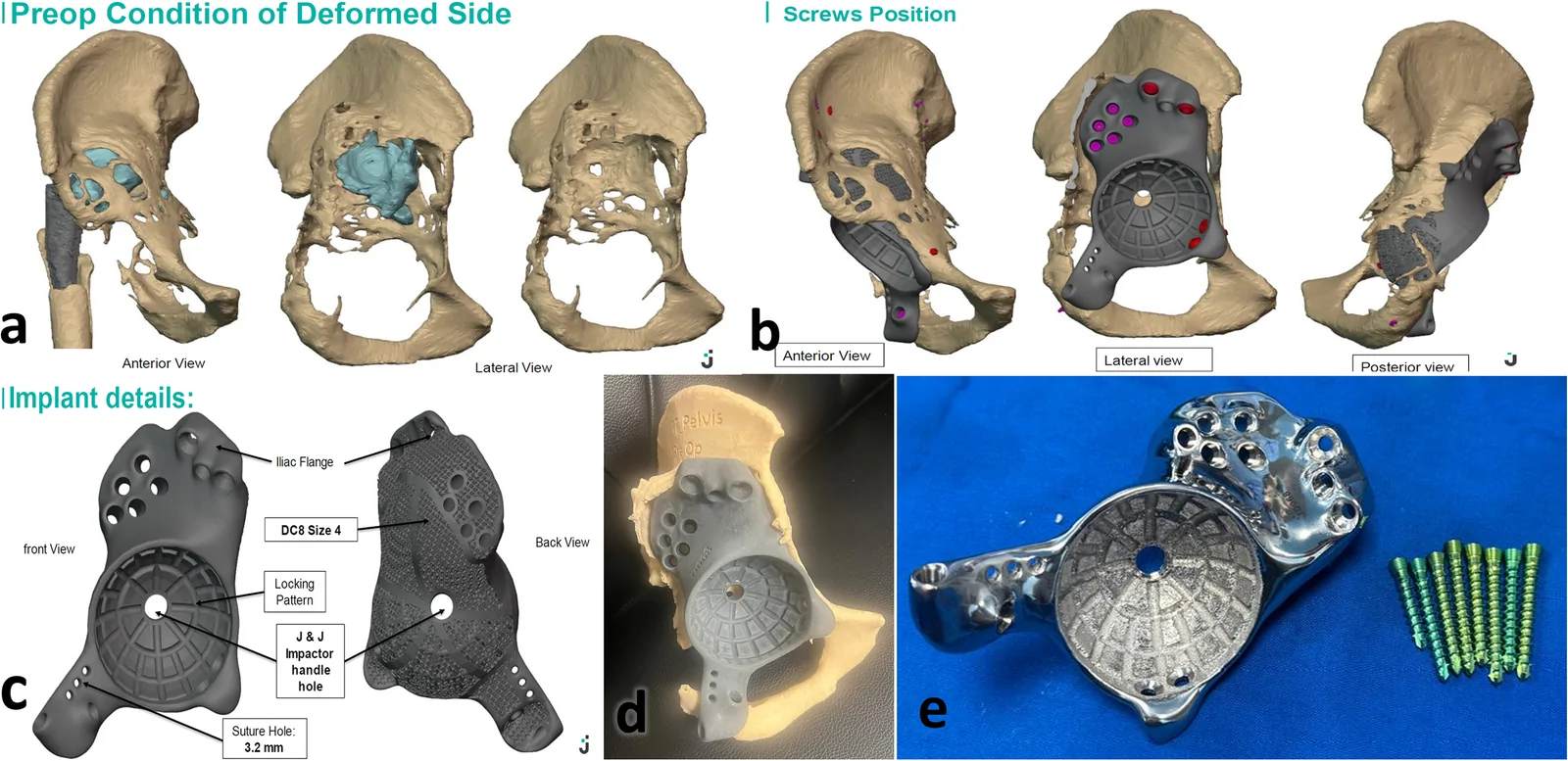
Segmentation and surgical planning. (a) 3D visualisation of the pelvis assessing the morphology of the remaining bone. (b) Schematic diagram showing positions of various locking screws with non-intersecting trajectories. (c) Design of the customized “implant”. (d) Life-size 3D-printed models of the custom implant and pelvis made of polylactic acid for intraoperative reference. (e) Custom 3D-printed implant having regions of trabecular titanium along with locking screws created using electron-beam additive manufacturing.

Revision surgery was performed via a posterior approach under general anaesthesia. Removal of the migrated components was technically demanding due to extensive fibrosis and proximal displacement. After debridement and preparation of viable host bone, the custom 3D-printed acetabular implant was secured using press-fit fixation supplemented with multiple locking screws placed along preplanned trajectories. A cemented polyethylene cup (Exeter X3 Rimfit Cup, Stryker Corp, Mahwah, NJ, USA) was implanted, and in the presence of extensive proximal femoral bone loss, a cemented proximal femoral replacement (xl-infinity by XLO, Ortho Life Systems Pvt. Ltd., New Delhi, India) was performed (Figures 3a–3c). Restoration of limb length and hip stability was confirmed intraoperatively. Postoperative radiographs demonstrated restoration of the hip COR and stable fixation at two-year follow-up (Figure 3d). The HHS, which evaluates pain, function, absence of deformity and range of movement, improved from 26 preoperatively to 70 at 2 years. Although this remained at the threshold between poor and fair, it represents a clinically meaningful improvement in hip function and pain following revision surgery [19].

**Figure 3:**
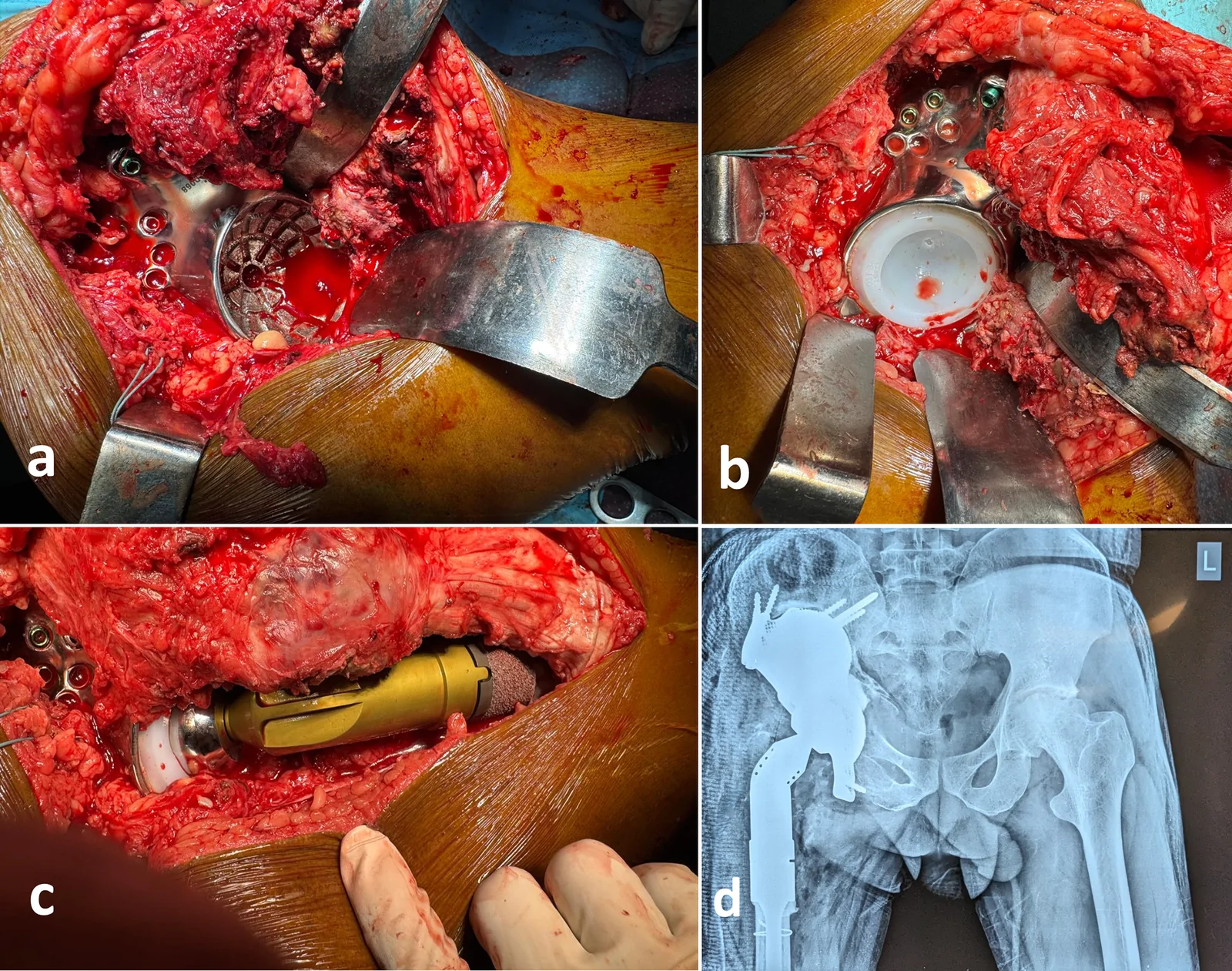
Intra-op photographs showing fixation of implants. (a) Fixation of the 3D-printed custom acetabular implant using locking screws. (b) Implantation of the polyethylene liner. (c) XL infinity^TM^ cemented proximal femoral prosthesis. (d) Follow-up radiograph at 2 years showing the custom 3D-printed acetabular implant and proximal femur prosthesis.

The second patient, a 64-year-old male with prior acetabular cage reconstruction done 12 years ago, presented with similar superior migration and periacetabular osteolysis (Figure 4a). Preoperative planning and implant design were done as described above. Intraoperative findings confirmed severe bone loss consistent with imaging (Figure 4b). The custom 3D-printed acetabular component was implanted using the same fixation principles, along with an Exeter X3 cemented cup and a revision monobloc stem (Reclaim, DePuy Ireland UC, Ireland) (Figure 4c). Postoperative radiographs demonstrated satisfactory positioning and restoration of acetabular alignment. At 1-year follow-up, patients’ HHS had improved from 47.85 to 81, corresponding to a good functional outcome (Figure 4d). This case further illustrates the reproducibility of the planning workflow and implant design across comparable defect morphologies.

**Figure 4:**
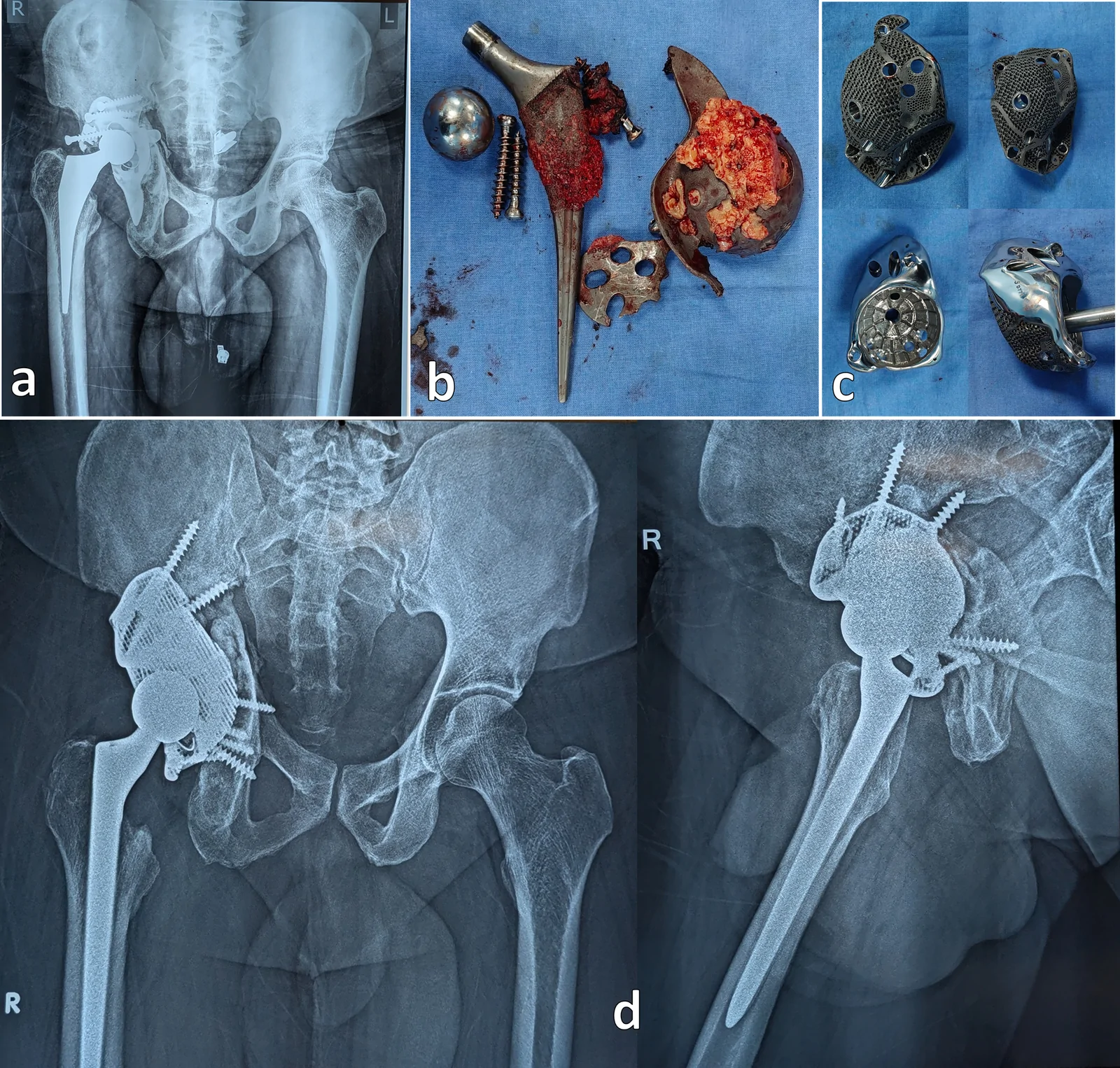
Radiographs and intraoperative photographs. (a) Anteroposterior (AP) radiograph of the pelvis showing a triflange cage and hip prosthesis. There is proximal migration of the acetabular component with surrounding osteolysis and loss of fixation of the screws. The femoral stem appears well fixed. (b) Intraoperative photograph showing bone loss during removal of the broken cage and femoral stem. (c) Custom 3D-printed acetabular implant to address acetabular bone loss. (d) Follow-up radiographs of the pelvis in AP and lateral views one year after surgery showing a well-fixed 3D-printed acetabular implant and femoral stem.

These cases demonstrate how patient-specific reconstruction can address complex acetabular defects that may be difficult to manage with standard modular implants. By tailoring implant geometry to the individual defect and optimising fixation strategy preoperatively, custom 3D-printed acetabular implants offer a rational solution in selected patients with extensive bone loss. In addition to meticulous surgical planning, ensuring the availability of cross-matched blood and blood components within a balanced transfusion protocol is essential for optimal postoperative outcomes, as significant intraoperative blood loss is anticipated in these cases.

## Discussion

Severe acetabular bone loss with superior migration of the failed acetabular component often precludes the use of conventional hemispherical cups or modular augments. Custom 3D-printed reconstruction addresses this challenge by restoring the hip COR while maximising implant–bone contact in areas of limited host bone. The incorporation of porous titanium surfaces facilitates biological fixation, whereas predefined screw trajectories and flanged extensions improve initial mechanical stability. Unlike earlier antiprotrusio cages, whose long-term success depended largely on osseointegration of the porous shell rather than the cage itself [10], patient-specific constructs are designed to maximise host bone contact and optimise fixation according to the individual defect morphology.

Achieving precise implant positioning and optimal contact between the bone and the implant surface is crucial for successful rTHA outcomes, as malpositioning can lead to instability and early failure [20]. Advanced imaging techniques, including computed tomography with metal artefact reduction, improve preoperative quantification of bone loss and facilitate more precise surgical planning. Importantly, minor early implant migration may occur before complete osseointegration without indicating construct failure, underscoring the need for longitudinal radiographic assessment rather than premature revision [11].

In the illustrative cases presented, restoration of limb length and hip biomechanics was achieved despite extensive bone loss. The reproducibility of the planning workflow across patients supports the practical applicability of patient-specific reconstruction. Compared with previously reported staged reconstructions using similar 3D-printed constructs, a single-stage approach may reduce cumulative surgical exposure and streamline care in non-infectious scenarios [21]. However, such decisions must remain individualised, and the functional improvements observed in our illustrative cases should be interpreted cautiously because both patients underwent concomitant femoral reconstruction. Therefore, postoperative HHS gains and perceived construct stability reflect the combined acetabular and femoral reconstruction rather than an isolated effect of the custom acetabular implant.

Existing literature provides further context. Systematic reviews have demonstrated satisfactory intermediate-term clinical and radiographic outcomes with custom acetabular implants in severe defects, although complication rates remain non-negligible [22]. Similarly, reports of custom 3D-printed acetabular components have shown favourable functional outcomes in selected patients with massive bone loss and PD [13, 17, 23]. Nevertheless, heterogeneity in implant design, surgical technique, and follow-up duration limits direct comparison across studies.

Custom 3D-printed acetabular reconstruction is not without limitations. This approach requires advanced imaging, specialised planning infrastructure, and coordination with manufacturing teams, which may increase cost and production time compared with off-the-shelf modular systems. Manufacturing quality also remains important, as micro-CT studies have identified occasional structural voids within some commercially available 3D-printed acetabular cups that may theoretically act as stress concentrators, highlighting the need for robust quality assurance [24].

Current preoperative planning, as described in these cases, is limited by the manual CT image segmentation, which can be labour-intensive, time-consuming and subject to interobserver variability, particularly as clinical demands for rTHA continue to rise. Artificial Intelligence offers a potential solution by automating segmentation of the residual host bone, osteolytic defects and implant artefacts, thereby accelerating 3D model generation and improving planning consistency. Zhang et al. [25] reported that AI-assisted preoperative planning achieved a greater than 85% conformity rate between planned and implanted acetabular and femoral components, suggesting that AI-assisted planning may improve implant templating and reduce prosthetic mismatch, thereby potentially improving implant positioning and reducing technical errors during surgery. Beyond segmentation, AI-driven biomechanical modelling may assist in optimising implant geometry, porous surface distribution, flange design, and screw trajectories prior to manufacture, providing a more efficient pathway from CT acquisition to custom implant production.

The benefits of AI-assisted planning may be further enhanced through integration with intraoperative technologies. Robotic-assisted systems, including MAKO and TSolution-One, have demonstrated improved accuracy and reproducibility of component positioning in primary THA [26]. Although their application in revision arthroplasty remains limited due to distorted anatomy and severe bone loss, combining robotic guidance with AI-assisted planning and augmented reality navigation may improve reproduction of the planned implant position during complex acetabular reconstruction. Following surgery, AI-driven analytics, wearable technologies, and sensor-enabled implants have the potential to continuously monitor gait, range of motion, implant loading, and micromotion, enabling earlier detection of mechanical failure and more personalised rehabilitation strategies [27, 28]. Nevertheless, AI algorithms are frequently developed using relatively homogeneous datasets, potentially limiting their generalisability across diverse patient populations and implant systems. In addition, infrastructure requirements, licensing costs, regulatory approval, and integration into existing surgical workflows may limit widespread adoption [29].

Despite these limitations, patient-specific 3D-printed acetabular implants remain an important reconstructive option when biologic and modular prosthetic techniques cannot adequately address severe acetabular bone loss. Published series have consistently demonstrated favourable short- to long-term clinical and radiographic outcomes, with improvements in HHS, low rates of radiographic loosening, and implant survivorship approaching 95% in selected cohorts [13, 16–18, 23, 30]. Nevertheless, the available evidence is derived predominantly from retrospective case series with heterogeneous implant designs. Future prospective multicentre studies using standardised implant designs, comparative cohorts and longer follow-up are required to establish the durability, cost-effectiveness, and comparative effectiveness of custom 3D-printed acetabular reconstruction.

## Conclusion

Reconstruction of severe acetabular bone loss in revision THA has evolved from biologic grafting to modular porous metal constructs and patient-specific 3D-printed acetabular components. By enabling anatomically matched reconstruction, restoration of the hip COR, and optimised fixation in compromised host bone, custom implants represent an important advance for managing complex acetabular defects when conventional techniques are insufficient. Although current evidence demonstrates favourable short- to long-term clinical and radiographic outcomes, it remains limited by predominantly retrospective studies with heterogeneous implant designs. Emerging technologies, including AI-assisted planning, robotic guidance, and sensor-enabled implants, may further improve implant design, surgical precision, and postoperative monitoring, but require prospective validation before routine clinical implementation.

## Data Availability

This article has no associated data generated and/or analysed.
